# Identification of diagnostic biomarkers and immune cell infiltration in coronary artery disease by machine learning, nomogram, and molecular docking

**DOI:** 10.3389/fimmu.2024.1368904

**Published:** 2024-04-02

**Authors:** Xinyi Jiang, Yuanxi Luo, Zeshi Li, He Zhang, Zhenjun Xu, Dongjin Wang

**Affiliations:** ^1^ Department of Cardio-Thoracic surgery, Nanjing Drum Tower Hospital, Chinese Academy of Medical Sciences & Peking Union Medical College, Peking Union Medical College Graduate School, Nanjing, China; ^2^ Peking Union Medical College Hospital, Chinese Academy of Medical Sciences & Peking Union Medical College, Beijing, China; ^3^ Department of Cardio-Thoracic Surgery, Nanjing Drum Tower Hospital, The Affiliated Hospital of Nanjing University Medical School, Nanjing, China

**Keywords:** coronary artery disease, diagnostic biomarkers, machine learning, nomogram, immune cell infiltration, molecular docking

## Abstract

**Background:**

Coronary artery disease (CAD) is still a lethal disease worldwide. This study aims to identify clinically relevant diagnostic biomarker in CAD and explore the potential medications on CAD.

**Methods:**

GSE42148, GSE180081, and GSE12288 were downloaded as the training and validation cohorts to identify the candidate genes by constructing the weighted gene co-expression network analysis. Functional enrichment analysis was utilized to determine the functional roles of these genes. Machine learning algorithms determined the candidate biomarkers. Hub genes were then selected and validated by nomogram and the receiver operating curve. Using CIBERSORTx, the hub genes were further discovered in relation to immune cell infiltrability, and molecules associated with immune active families were analyzed by correlation analysis. Drug screening and molecular docking were used to determine medications that target the four genes.

**Results:**

There were 191 and 230 key genes respectively identified by the weighted gene co-expression network analysis in two modules. A total of 421 key genes found enriched pathways by functional enrichment analysis. Candidate immune-related genes were then screened and identified by the random forest model and the eXtreme Gradient Boosting algorithm. Finally, four hub genes, namely, CSF3R, EED, HSPA1B, and IL17RA, were obtained and used to establish the nomogram model. The receiver operating curve, the area under curve, and the calibration curve were all used to validate the accuracy and usefulness of the diagnostic model. Immune cell infiltrating was examined, and CAD patients were then divided into high- and low-expression groups for further gene set enrichment analysis. Through targeting the hub genes, we also found potential drugs for anti-CAD treatment by using the molecular docking method.

**Conclusions:**

CSF3R, EED, HSPA1B, and IL17RA are potential diagnostic biomarkers for CAD. CAD pathogenesis is greatly influenced by patterns of immune cell infiltration. Promising drugs offers new prospects for the development of CAD therapy.

## 1 Introduction

Coronary artery disease (CAD) is an atherosclerotic disease caused by a variety of environmental, genetic, and other risk factors. When aberrant lipid metabolism, endothelial injury, or changed hemodynamics release proinflammatory substances, inflammatory cells in the circulating pool congregate, penetrate the coronary artery intima, and interact with vascular smooth muscle cells (1, 2). Under the influence of colony-stimulating substances, infiltrating monocytes develop into macrophages and phagocytose lipoproteins, becoming foam cells. Eventually, an area made up of a fibrous cap, apoptotic necrotic cells, cholesterol crystals, and other extracellular material forms because of the latter’s reduced capacity to migrate, grow, mature, and die in the intima. This is also accompanied by remodeling of the arterial wall (3–6). Plaque causes varying degrees of narrowing of the arterial lumen. When the plaque ruptures, the clot moves with the blood flow and if a blockage is formed, it causes ischemia in the subsequent area, and in severe cases, in other words, myocardial infarction (MI) (7, 8). It was recorded that more than 700,000 people experience an AMI- or CAD-related death per year (9).

The genome-wide association studies (GWAS) identified 33 genetic variants associated with increased risk of CAD (10). A meta-analysis showed that the G487A polymorphism in the ALDH2 gene was significantly associated with an increased risk of CAD in the Chinese population (11). Rare variant association studies (RVAS) indicated that at least 11 genes have been shown to alter the risk of coronary heart disease, including cholesterol metabolism (LDLR, PCSK9, NPC1L1), triglyceride metabolism (APOA5, APOC3, LPL, ANGPTL4, ANGPTL3), Lp(a) (LPA), non-high-density lipoprotein (NHDL) cholesterol (ASGR1), and blood pressure-related variants (SVEP1). Ferroptosis, cuproptosis, N6-methyladenosine (m6A), and other modalities are simultaneously influencing CAD progression (12–14). Dai et al. pointed out that the imbalance of the flora structure is related to the disorder of lipid metabolism and immunity, which promotes the occurrence of CAD. In addition, a wide range of circulating metabolites can promote or inhibit vascular atherosclerosis and plaque stability by affecting intestinal barrier, lipid transport, and secretion of inflammatory factors (15).

The investigation of alterations in disease-related gene expression and the identification of such potential genes with the goal of developing novel diagnostic and therapeutic strategies are both made possible using the microarray technique. Gene expression levels are an essential indicator for making a preliminary diagnosis and can reveal the severity of several illnesses. The most pertinent module with the clinical characters can be chosen using the weighted gene co-expression network analysis (WGCNA) approach. Machine learning algorithms have shown considerable promise in examining the underlying relationship of high-dimensional data using either supervised or unsupervised methodologies. The impact of diagnostic biomarkers may be further evaluated using a nomogram, which was verified by ROC and AUC. Molecular docking and drug screening may offer new therapies on the CAD. Through each of these methods, we investigate possible CAD target genes in this article, as well as the function of immune cells in the illness, in order to fill the gap in CAD research.

## 2 Materials and methods

### 2.1 Data collection and preprocessing

Three gene expression profile datasets (GSE42148, GSE180081, and GSE12288) were accessed from the Gene Expression Omnibus (GEO) database (https://www.ncbi.nlm.nih.gov/geo/) by the National Center for Biotechnology Information (NCBI). The mRNA expression profiling dataset GSE42148 was downloaded as the training cohort, which was annotated by GPL13607. GSE42148 contains 24 samples (13 samples with angiographically confirmed CAD and 11 population-based asymptomatic controlled samples). All samples were obtained from the enrolled patients’ whole blood. Single-molecule sequencing of RNA (RNAseq) GSE180081 was obtained as the validation cohort annotated by GPL14761. A total of 96 samples including 48 with LOW coronary stenosis and 48 with mid and severe (MID+) coronary stenosis were contained in GSE180081 annotated by GPL14761. GSE12288 was utilized as a validation cohort by 110 CAD samples and 112 control samples annotated by GPL96. For the problem of multiple probes corresponding to the same gene, the average value was retained using the limma package.

### 2.2 Construction of the weighted gene co-expression network analysis

WGCNA sought to identify co-expressed gene modules and investigate the association between the gene network and the phenotype of interest. Using the WGCNA package, the co-expression network of all genes in the GSE42148 dataset was built. Based on the weighted correlation coefficient, genes were classified into multiple modules according to their shared expression patterns, and the colors reflected the various modules. We then selected the two modules with the strongest correlations for subsequent analysis. The correlation between gene expression and trait >0.2 and the correlation between gene expression and module >0.8 were used as criteria for screening, and a total of 421 genes were finally identified.

### 2.3 Functional enrichment analysis

Functional enrichment analysis was applied to identify the likely function of potential targets using the clusterProfiler package. With the Gene Ontology (GO) database, we can learn about the functional relationship of target genes at biological process (BP), cellular component (CC), and molecular function (MF) levels. Based on the annotation of the function of candidate genes themselves, we can find various pathways in which the target gene is involved in the human body through the Kyoto Encyclopedia of Genes and Genomes (KEGG) database. The standard of p-value and the adjusted p-value were both set as 0.05.

### 2.4 Screening of the candidate immune-related genes

The Immunology Database and Analysis Portal (ImmPort) database (https://www.immport.org/) is a repository for storing and sharing a wide range of immune-related resources. All the immune-related genes were downloaded and then intersected with the former 421 genes using the Venn package. We then validated the intersected genes in GSE42148 and visualized them by violin plots. Only six immune-related genes (IRGs) exhibited the same trend as their own module.

### 2.5 Identification of the diagnostic biomarkers by machine learning algorithms

The six IRGs were trained by these two machine learning algorithms. The Random Forest (RF) model was formed by multiple decision trees. Each decision trees may give a vote, with the most votes ultimately being the final model predictions. Initial screening was performed using the training cohort: 500 trees were set as the total number of decision trees; 10-fold cross-validation and importance scoring were carried out, sorting and visualizing based on the least out-of-bag (OOB) error estimate; and finally the top five genes were selected. The eXtreme Gradient Boosting (XGBoost) algorithm supports regression and classification predictive modeling problems. It not only greatly reduces computation time but also improves prediction accuracy. After importing the data from training cohort, the model was stabilized at a maximum of 17 iterations (nround). Based on the gene significance score, the top five genes were selected. Both selected genes were intersected. Finally, four hub genes were chosen and recognized as diagnostic biomarkers for further correlation analysis.

### 2.6 Construction of nomogram

Four diagnostic biomarkers were used to establish the nomogram model. The receiver operating characteristic (ROC) curve and its area under curve (AUC) were both used to determine the utility and accuracy of the nomogram model based on the four genes. Calibration curve indicated that the model had a good prediction value.

### 2.7 Correlation analysis between diagnostic biomarkers and infiltrating immune cells

We used the CIBERSORTx online website (https://cibersortx.stanford.edu/) to further explore the correlations between diagnostic biomarkers and the 29 infiltrating immune cells in CAD groups, which was presented through result visualization. The p-value < 0.05 was recognized to be significantly important.

### 2.8 Correlation analysis among three families of immune-related active molecules

In order to investigate the significance of the four hub genes for the immune system even more, we conducted a correlation study between them and key genes from three families of immune-related active molecules.

### 2.9 Gene set enrichment analysis

Gene set enrichment analysis (GSEA) was analyzed on the basis of the full dataset, using a specific dataset to identify whether there were differences between the two groups and whether specific pathways were enriched between groups. The whole CAD samples were selected and grouped into high and low expression groups using the median expression values of the four genes and then analyzed by the GSEA enrichment. There were 7,233 hallmark gene sets downloaded from the Molecular Signatures Database (MSigDB) online (https://www.gsea-msigdb.org/gsea/msigdb) as the reference gene set, and clusterProfiler and GseaVis packages were used for gene enrichment and visualization.

### 2.10 Drug screening and molecular docking

Drug screening and molecular docking were used to determine medications that target the four genes. First, we searched the Comparative Toxicogenomics Database (CTD) database (https://ctdbase.org/) for a list of small-molecule compounds that might either up- or downregulate the expression of the relevant genes. From the Chemical Entities of Biological Interest (ChEBI) database (https://www.ebi.ac.uk/), we subsequently retrieved the structures of these small-molecule compounds. Furthermore, the AlphaFold Protein Structure Database (https://alphafold.ebi.ac.uk/) provided us with the anticipated protein structures encoded by the aforementioned genes. AutoDock Vina was used to carry out molecular docking ultimately.

## 3 Results

### 3.1 Identification of key modules and genes in WGCNA

**Figure 1:**
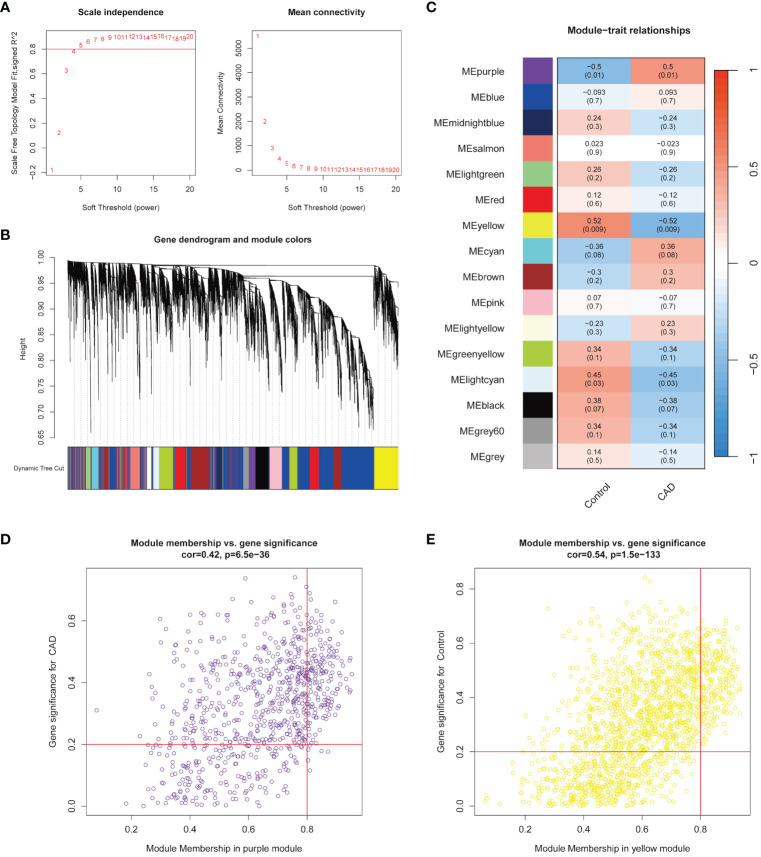
Identification of modules highly correlated with CAD. **(A)** Topology analysis and mean connectivity analysis for a series of soft threshold powers. **(B)** Module clustering dendrogram assigned with different module colors. **(C)** Heatmap exhibited the relevance of different color modules to CAD. **(D)** In the CAD group, the most highly correlated purple module was selected to present the correlation between module membership and gene significance. **(E)** In the control group, the most highly correlated yellow module was selected to present the correlation between module membership and gene significance.

The Pearson’s correlation coefficient was applied, and all samples in GSE42148 were well clustered. The scale-free topology criterion with R^2^ = 0.8 and the soft threshold β = 5 were set (**Figure 1A**). The dendrogram of all genes was clustered based on a topological overlap matrix (TOM, **Figure 1B**). Each branch in the clustering tree represented one gene, and each color represented one module. A total of 16 modules were identified and visualized as a module-trait heatmap to describe the correlation between module genes and CAD (**Figure 1C**). After calculating the corresponding correlation coefficient and p-value, the purple module showed the strongest relationship with the CAD group (cor = 0.5 and p = 0.01) and the yellow module showed the same with the control group (cor = 0.52 and p = 0.009). These two modules were then selected for the further analysis. The module membership (MM) and gene significance (GS) of the purple and yellow modules showed a significant association (**Figures 1D, E**). There were 191 and 230 key genes identified in the two modules, respectively, with the standard of geneTraitSignificance > 0.2 and geneModuleMenbership > 0.8 (**Supplementary Tables 1, 2**).

### 3.2 Functional pathway analysis

**Figure 2:**
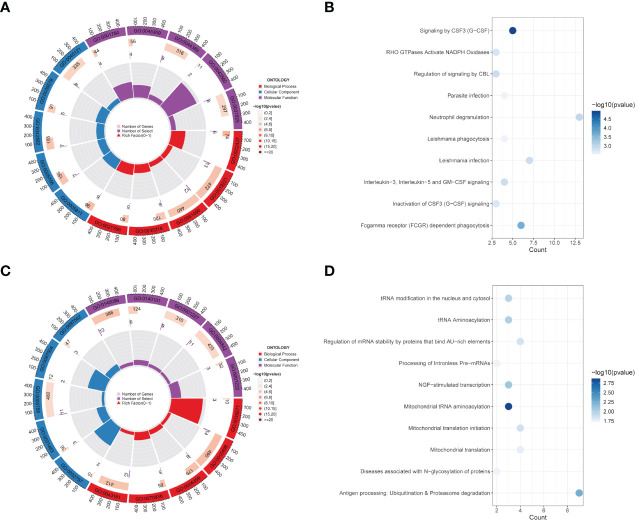
Functional enrichment analysis of modules. **(A, B)** GO enrichment analysis **(A)** and KEGG pathway enrichment analysis **(B)** of 191 genes belong to the purple module in the CAD group. **(C, D)** GO analysis result **(C)** and KEGG enrichment analysis result **(D)** for 230 genes corresponding to the yellow module in the control group.

GO and KEGG functional analyses were conducted for further explorations on the underlying roles on these key genes. GO enrichment analysis revealed multiple BP (including “hippocampus development,” “erythrocyte differentiation,” “regulation of DNA-binding transcription factor activity,” “cytokine-mediated signaling pathway,” and “inflammatory response to antigenic stimulus”), CC (“membrane raft,” “azurophil granule lumen,” “azurophil granule,” “primary lysosome,” and “lipid droplet”), and MF (“phosphotyrosine residue binding,” “protein phosphorylated amino acid binding,” “ubiquitin-like protein ligase binding,” “insulin receptor substrate binding,” and “ubiquitin protein ligase binding”) levels enriched in purple module and top five of each levels were visualized in **Figure 2A**. KEGG enrichment analysis again enriched various pathways, including “Signaling by CSF3 (G-CSF),” “RHO GTPases Activate NADPH Oxidases,” “Regulation of signaling by CBL,” “Parasite infection,” “Neutrophil degranulation,” “Leishmania phagocytosis,” “Leishmania infection,” “Interleukin-3. Interleukin-5 and GM-CSF signaling,” “Inactivation of CSF3 (G-CSF) signaling,” and “Fcgamma receptor (FCGR) dependent phagocytosis” (**Figure 2B**). **Figures 2C, D** show GO and KEGG enrichment analyses in yellow module same steps as above.

### 3.3 Screening and validating on IRGs

**Figure 3:**
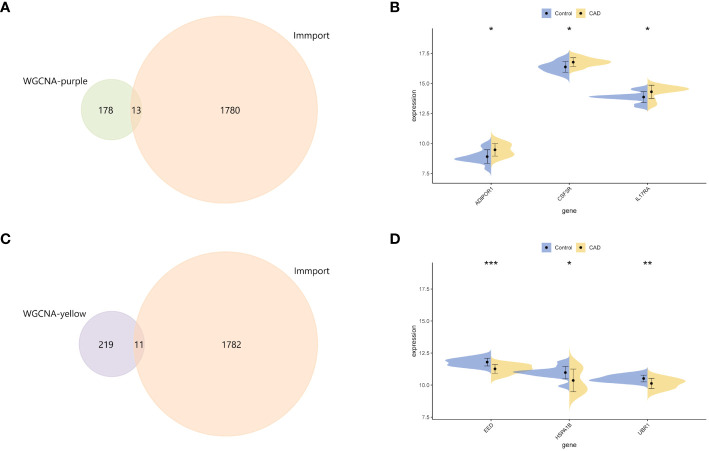
Identification of central genes. **(A)** The intersection Venn diagram of immune genes from the ImmPort database and key genes of the purple module. **(B)** Split violin plots showed the mRNA expression level of ADIPOR1, CSF3R, and IL17RA. **(C)** The Venn diagram revealed the key genes from the intersection between immune-related genes and core genes from the yellow module. **(D)** Split violin plots showed the mRNA expression level of EED, HSPA1B, and UBR1. *p < 0.05, **p < 0.01, ***p < 0.001.

To further discover more information on IRGs, 191 key genes in purple module and 230 key genes in yellow module were both intersected with 1,793 immune genes downloaded from the ImmPort database (**Figures 3A, C**). There were 13 and 11 IRGs obtained and examined their expression levels in validation dataset, respectively. Only three genes (ADIPOR1, CSF3R, and IL17RA) upregulated in purple module and three genes (EED, HSPA1B, and UBR1) downregulated in yellow module (**Figures 3B, D**). These six IRGs were then selected into further analyses.

### 3.4 Machine learning for diagnostic biomarkers

**Figure 4:**
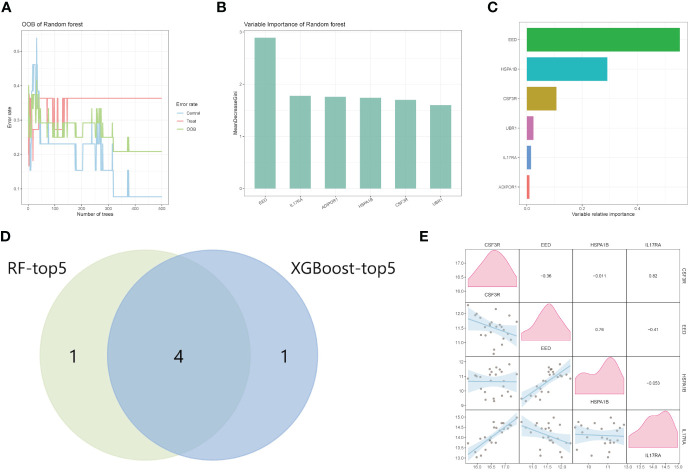
Machine learning for hub genes. **(A, B)** Random Forest analysis of HIGs. **(C)** The ranking of candidate genes based on the variable relative importance using the XGBoost algorithm. **(D)** The intersection Venn diagram of RF-top five genes and XGBoost top five genes. **(E)** The correlation analysis between GSF3R, EED, HSPA1B, and IL17RA. RF, random forest.

We first established the RF model using six IRGs (**Figure 4A**). In descending order of variable importance were EED, IL17RA, ADIPOR1, HSPA1B, CSF3R, and UBR1, respectively (**Figure 4B**). The XGBoost algorithm was then used to identify the hub genes. The variables with varying degrees of relative importance were EED, HSPA1B, CSF3R, UBR1, IL17RA, and ADIPOR1, in descending order (**Figure 4C**). Top five genes of each algorithm were intersected to determine the hub genes. Results showed that four hub genes, CSF3R, EED, HSPA1B, and IL17RA, were obtained and recognized as the diagnostic biomarkers for CAD (**Figure 4D**). The correlation analysis of expression levels of four hub genes is shown in **Figure 4E**.

### 3.5 Construction of the nomogram model for CAD prediction

**Figure 5:**
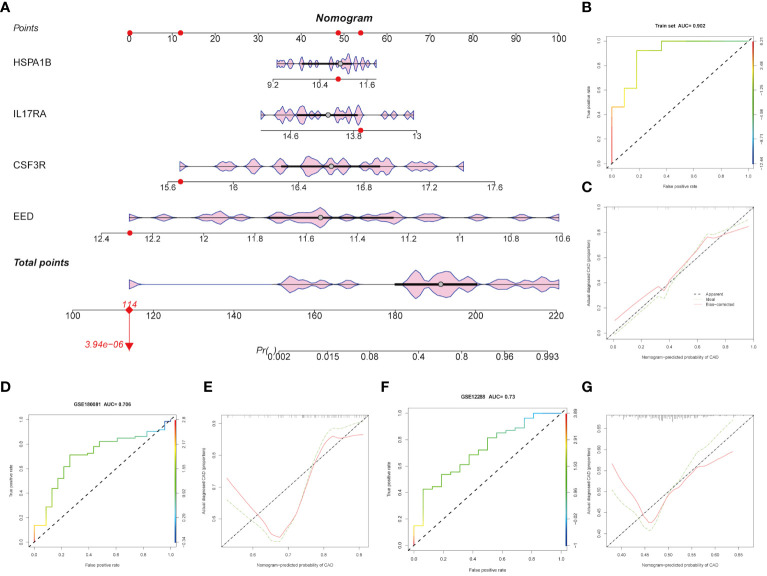
Construction and validation of the hub genes. **(A)** Diagnostic nomogram of the four hub genes. **(B-G)** ROC curve and nomogram calibration curves in the training cohort and external validation cohort.

Based on the four hub genes mentioned above (CSF3R, EED, HSPA1B, and IL17RA), we constructed a nomogram model to predict CAD. As **Figure 5A** shows, our model performed well in CAD prediction. We executed an ROC analysis to further evaluate the nomogram model’s predictable power and the AUC was 0.902 (**Figure 5B**). The calibration curve also showed good predictive value (**Figure 5C**). GSE180081 and GSE12288 datasets were validated the utility and accuracy of the nomogram model. Both ROC indicated good predictive value, and the AUC values were 0.706 and 0.73, respectively (**Figures 5D, F**). Calibration curves also showed good predictive value (**Figures 5E, G**).

### 3.6 Correlations with immune cells

**Figure 6:**
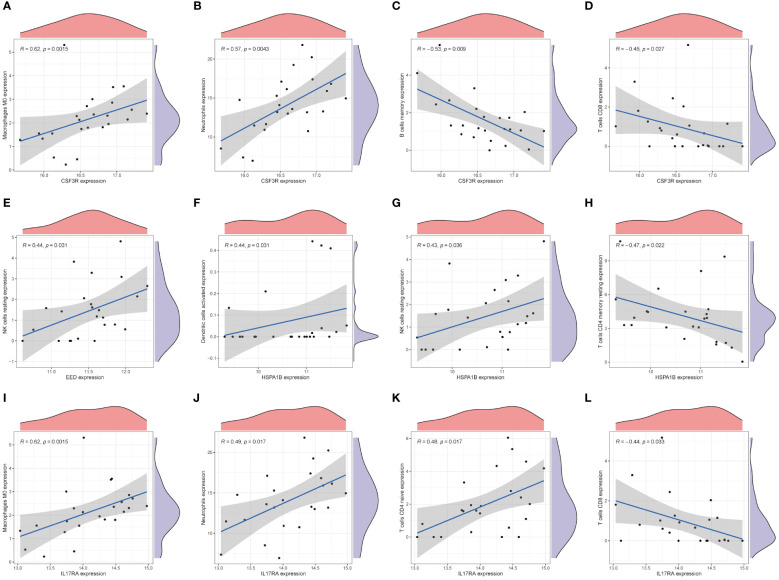
Immune characteristics of hub genes. The correlation between the infiltration of immune cells and mRNA expression level of core genes, CSF3R **(A-D)**, EED **(E)**, HSPA1B **(F-H)**, and IL17RA **(I-L)**.

We used CIBERSORTx online websites to calculate and predicted the abundance of hub genes among immune cells. As **Figures 6A, B** show, CSF3R exhibited positive relationships with M0 macrophages (R = 0.62, p = 0.0015) and neutrophils (R = 0.57, p = 0.0043). Instead, memory B cells (R = −0.53, p = 0.009) and CD8+ T cells (R = −0.45, p = 0.027) negatively related with CSF3R (**Figures 6C, D**). Only resting NK cells (R = 0.44, p = 0.031) showed three families played pivotal roles in CAD a positive relationship with EED (**Figure 6E**). For the HSPA1B gene, activated dendritic cells (R = 0.44, p = 0.031) and resting NK cells (R = 0.43, p = 0.036) indicated positive correlations whereas CD4+ T memory resting cells (R = -0.47, p = 0.022) indicated negative (**Figures 6F–H**). Moreover, M0 macrophages (R = 0.62, p = 0.0015), neutrophils (R = 0.49, p = 0.017), and CD4+ T naive cells (R = 0.48, p = 0.017) all showed positive correlations with IL17RA (**Figures 6I–K**). CD8+ T cells negatively correlated with IL17RA, in contrast (**Figure 6L**).

### 3.7 Immune-related active molecules of three families played pivotal roles in CAD

**Figure 7:**
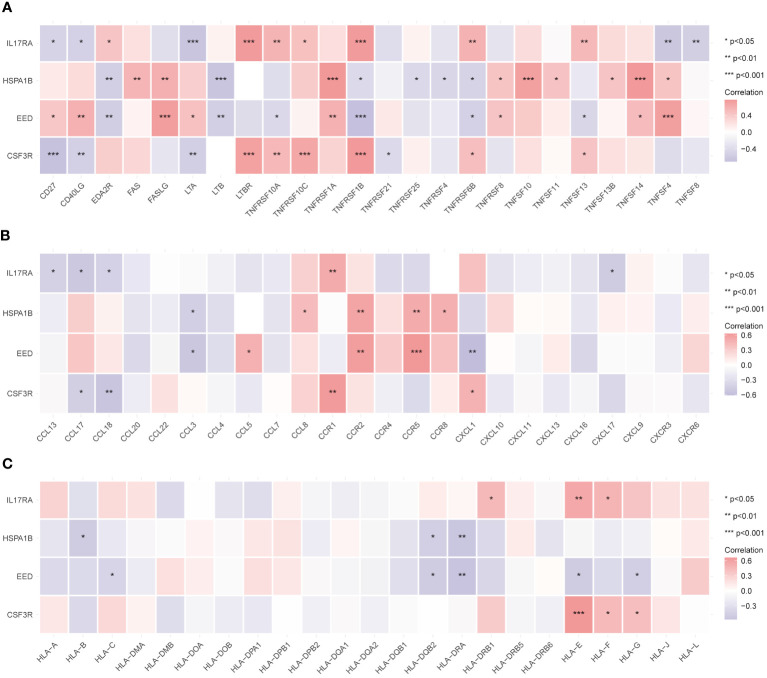
Relationship between immune-related active molecules and hub genes. Correlation analysis between the TNF family **(A)**, chemokine family **(B)**, and HLA family **(C)** members and the hub genes. *p < 0.05, **p < 0.01, ***p < 0.001.

We examined the correlations with the three main immune families’ compounds to delve even further into the probable immunological roles into four hub genes. As **Figure 7A** shows, most molecules (especially TNFRSF1B and TNFRSF6B) in the tumor necrosis factor (TNF) family had strong relationships with four hub genes. Members in the chemokine family such as CCR1, CCR2, CCR5, and CXCL2 exhibited significantly important correlations with hub genes (**Figure 7B**). Similarly, human leukocyte antigen (HLA) family members such as HLA-DRA, HLA-E, and HLA-F correlated with hub genes (**Figure 7C**). To sum up, biological pathways including these three families might play pivotal roles in CAD generation and progression.

### 3.8 GSEA analysis in the CAD group

**Figure 8:**
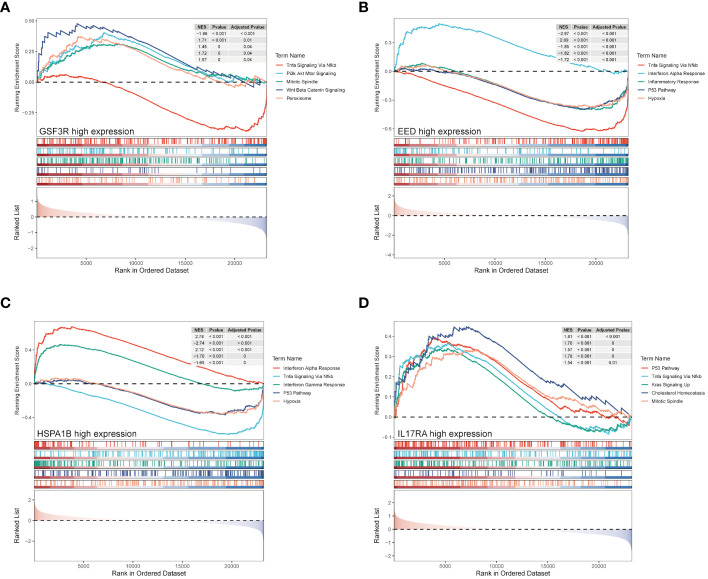
GSEA enrichment analysis of hub genes. GSEA analysis result of hub genes from CSF3R high- and low-groups **(A)**, EED high- and low-groups **(B)**, HSPA1B high- and low-groups **(C)**, and IL17RA high- and low-groups **(D)**.

Based on the median expression value of four hub genes, we divided the CAD group into high and low groups respectively and performed a GSEA. **Figures 8A–D** represent GSF3R, EED, HSPA1B, and IL17RA high expression groups’ results, respectively. TGSF3R high expression group was mostly enriched in “TNF-α signaling *via* NF-κB,” “PI3K/AKT/mTOR signaling,” “Mitotic spindle,” “Wnt/β-catenin signaling,” and “Peroxisome” (**Figure 8A**). The EED high expression group was mostly enriched in “TNF-α signaling *via* NF-κB,” “Interferon alpha response,” “Inflammatory response,” “P53 pathway,” and “Hypoxia” (**Figure 8B**). The HSPA1B high expression group was mostly associated with “Interferon alpha response,” “TNF-α signaling *via* NF-κB,” “Interferon gamma response,” “P53 pathway,” and “Hypoxia” (**Figure 8C**). IL17RA was mostly associated with “P53 pathway,” “TNF-α signaling *via* NF-κB,” “KRAS signaling up,” “Cholesterol homeostasis,” and “Mitotic spindle” (**Figure 8D**).

### 3.9 Potential drug screening and molecular docking for hub genes

**Figure 9:**
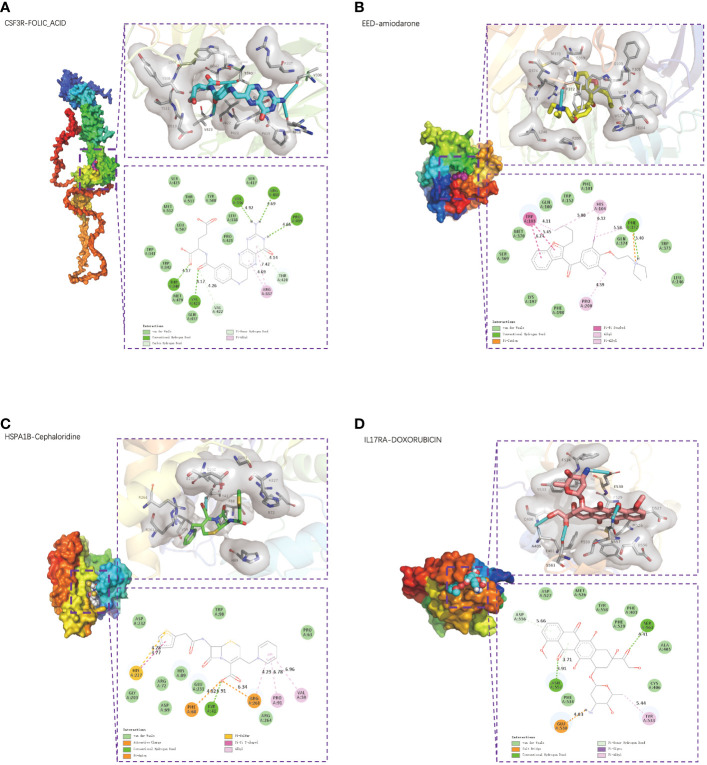
Small-molecule drug molecular docking. The 3D structure diagrams of molecular docking presented the small-molecule drug targeting CSF3R **(A)**, EED **(B)**, HSPA1B **(C)**, and IL17RA **(D)** respectively. The 3D structure diagrams presented the molecular docking result of CSF3R with folic acid (A), EED with amiodarone **(B)**, HSPA1B with cephaloridine **(C)**, and IL17RA with doxorubicin **(D)**.

Using the CTD database, we searched possible drugs that could upregulate EED or HSPA1B expression and downregulate CSF3R or IL17RA expression, respectively, since CSF3R and IL17RA are risk factors and EED and HSPA1B are protective factors in CAD. **Supplementary Table 3** displays many medications together with their binding energies. Particularly, the most stable combination ability was revealed by CSF3R-FOLIC-ACID (−8 kcal/mol, **Figure 9A**), EED-amiodarone (−8.2 kcal/mol, **Figure 9B**), HSPA1B-cephaloridine (−6.9 kcal/mol, **Figure 9C**), and IL17RA-DOXORUBICIN (−7.4 kcal/mol, **Figure 9D**).

## 4 Discussion

A complex, chronic, and ever-evolving inflammatory condition called coronary atherosclerosis is defined by remodeling of the coronary arteries, which blocks the heart’s ability to get oxygen. Acute coronary syndromes, stable angina, heart failure, sudden cardiac death, and other clinical manifestations are just a few of its many clinical manifestations. MI, which is driven by both genetic and environmental susceptibility and the interactions between these factors, has been identified as the leading cause of death worldwide. Owing to aging demography, hypertension, diabetes, obesity, unhealthy lifestyles, and environmental changes after prosperous economic promotions, the incidence of CAD has been tremendously increased worldwide (16, 17). For decades, hundreds of genetic loci that are strongly associated with CAD or CAD-related traits have been discovered by GWAS, but only individuals who inherit a combination of several variants are most at risk of developing the disease (10, 18, 19). When coronary atherosclerosis develops an unstable phase with the systemic inflammatory system reacting vigorously, MI may happen. Unstable atherosclerotic plaques, characterized by large lipid pools, high levels of macrophages, a dearth of collagen, and thin caps of plaques may rupture and completely or incompletely occlude the artery due to thrombus formation, which results in an ST elevation MI (STEMI) or a non-STEMI, respectively (20, 21). So far, research on CAD needs further advances. This may allow for a better understanding of how CAD develops and affects our immune system and on the other hand unravel the deeper meaning behind the disease.

In this study, we developed explorations on the diagnostic gene markers of CAD, validation on diagnostic model, excavations on immune cell infiltration and immune-related active molecules, and discoveries on promising meditations therapy were investigated. WGCNA implements both weighted and unweighted correlation networks, focuses on the underlining relationships between genes and clinical information, and then determines the most relevant module. By constructing a WGCNA co-expression network, the purple and yellow modules were chosen. Limited standards further selected 191 and 230 key genes in two modules, respectively. GO and KEGG analyses revealed that most genes were enriched in multiple inflammation-related pathways. Screening for IRGs and selection of candidate genes were conducted. Moreover, two different machine learning algorithms, RF model and XGBoost, were utilized to determine the hub genes. The diagnostic nomogram model was established based on four hub genes. ROC and AUC as well as the calibration curves co-validated the accuracy of our model, which performed well in both training and validation cohorts. By comparing genes’ expression to calculate the abundance of immune cell infiltration, we found that various immune cells, especially myeloid cells and NK/T cells, strongly correlated with hub genes. Immunocorrelation analysis further validated the strong relationship between CAD and the immune system. Molecular docking and identification of potential drug targets provided new prospects for the treatment of CAD.

CSF3R, also called colony-stimulating factor 3 receptor, encodes protein that is the receptor for colony-stimulating factor 3 (CSF3). CSF3R mutations can be detected in approximately 80% of patients with chronic neutrophilic leukemia (CNL), and CSF3R mutations are now used as specific diagnostic molecular markers for CNL and atypical chronic myeloid leukemia (aCML) (22). It is reported that proteins encoded by CSF3R may also function in cell surface adhesion and recognition processes. CSF3R signals through the JAK-STAT pathway, the non-receptor tyrosine kinase SYK, and the SRC family kinase LYN (23). Our study found that CSF3R expression was increased in the CAD group whereas the gene showed a positive association with neutrophils and macrophages, but negative with B and T cells. A previous study declared that an increased neutrophil-to-lymphocyte ratio (NLR) may act as a biomarker on adverse cardiovascular outcomes (24, 25). Interestingly, canakinumab (a selective anti-IL-1β monoclonal antibody) decreased neutrophil counts to lower NLR, which in turn decreased adverse endpoint events of cardiovascular disease with no discernible effect from lipid levels, which may indicate that neutrophil played a more vital role (26). Folic acid, also known as vitamin B9, must be formed through food intake and converted by gut microbes through digestion (27). As a coenzyme in the production of purines and pyrimidines, as well as in the conversion of one-carbon units and the methylation cycle, folic acid performs a variety of roles in the body (28). In recent years, a number of prospective and retrospective case–control studies have proved that a high level of plasma homocysteine is associated with risk cardiovascular disease (CVD) (29). Folic acid intake is effective in lowering homocysteine levels and thus reducing adverse cardiovascular events. There is proof that homocysteine increases the adhesion between neutrophils and endothelial cells (30). This phenomenon results in neutrophils moving across the endothelial layer while causing damage and shedding of endothelial cells. High doses of folic acid have also been found positively related with nitric oxide (NO), which may provide another prevention and treatment of CVD (31). On the other hand, monocytes develop into macrophages in response to colony-stimulating factors and form foam cells by phagocytizing low-density lipoprotein particles. Folic acid was found to protect against the progression of diabetic nephropathy in mice by inhibiting M1 macrophage polarization through inhibition of the nuclear factor-gene binding (NF-κB) signaling pathway in a mouse model (32). A study showed that a mixture of folic acid and methyl donors (e.g., folate, choline, and vitamin B12) reduced IL1B, TNF-α expression, and secretion of related proteins in THP-1 monocytes/macrophages. Furthermore, folic acid and choline reduced CCL2 mRNA levels, demonstrating that folic acid may help control the progression of chronic inflammation in inflammation-related diseases (33).

The polycomb-group (PcG) protein family induces cell differentiation through transcriptional repression. PcG proteins are part of two major transcriptional repression complexes, PRC1 and PRC2. PRC2 (polycomb repressive complex 2) is an important epigenetic modifying enzyme complex, whose catalytic activity depends on at least four subunits: the catalytic subunit EZH2 (zeste gene enhancer homolog 2), EED (embryonic ectodermal developmental protein), SUZ12 (zeste12 homolog 1 repressor 2), and RBBP46/48 (histone binding protein also known as RBBP7/4) (34). PRC2 functions as a transcription genetic regulator and is essential for several biological activities, including DNA repair and stem cell maintenance. Clinically, EZH2 firstly discovered involving in prostate cancer and is a major transcriptional target of the E2F-PRB tumor-suppressor pathway. Disrupting the interaction between EZH2 and EED may be helpful to tumor inhibition. In addition to demonstrating that epigenetically abnormal disorders can be reversed by epigenetic re-regulation, previous studies have shown that deletion of EED in cardiomyocytes contributes to the development of dilated cardiomyopathy, which may be related to the aberrant accumulation of H3K27ac (lysine residue 27 on histone H3 that undergoes acetylation) (35). **Figure 6E** shows that EED positively correlated with resting NK cells. JAK3 inhibitors were able to significantly reduce natural killer/T-cell lymphoma cell growth in an EZH2 phosphorylation-dependent manner (36). Study has shown increased apoptosis of NK cells in patients with CAD, suggesting that NK cells might play an inhibitory role in the disease (37). Amiodarone is a class III medication that primarily functions as an antiarrhythmic by blocking cardiac potassium channels and voltage-gated sodium channels (38). Amiodarone can also selectively dilate the coronary artery while lowering peripheral resistance and slowing the heart rate through non-competitive inhibition of adrenergic receptors (39). Han et al. indicated that amiodarone resulted in an increased expression of EED (40). Amiodarone-treated neural stem cells (NSCs) significantly upregulated multiple transcription factors that were involved in TNF-α receptor-mediated apoptosis through the formation of the DNA-binding complex AP-1. From **Figure 7A**, which further illustrates the strong association between EED and the TNF family, we hypothesize that amiodarone may affect EED expression through its modulation of the TNF molecular pathway, which is involved in the progression of CAD. A more in-depth discussion is needed on the relationship between amiodarone and EED.

HSPA1B (heat shock protein family A member 1B), one of the isoforms in HAP70, is a stress-inducible molecular chaperone whose key role involves the refolding and degradation of polypeptides, forming complexes with other heat shock proteins (e.g., HSP90), stabilizing existing proteins against aggregation, and mediating the folding of newly translated proteins in the cytoplasm and organelles (41). In addition, HSP70 secreted into the extracellular matrix leads to the upregulation of the expression of proinflammatory factors such as TNF by stimulating signaling cascades (42). This confirms that HSPA1B is closely related to the TNF family, as shown in **Figure 7A**. We found that HSPA1B may be correlated with T cells. A single-cell pan-cancer study last year detected a novel CD4+/CD8+ T-cell subtype characterized by a high expression of heat shock protein genes such as HSPA1A, HSPA1B, and other stress-response-related genes, known as the stress response (T_STR_) (43). Meanwhile, NF-κB expression was upregulated in this cell subset, and the signaling pathway mediated by NF-κB plays a key role in regulating the cellular stress response. Results also showed that T_STR_ cells were in low abundance or undetectable in healthy/non-involved tumor tissues, whereas they were highly enriched in primary tumors or metastases, mainly localized in the tumor hypoxic microenvironment. In light of this, it is suggested that HSPA1B may function by activating several signaling pathways and contribute significantly to the development of CAD under specific conditions. Cephaloridine is a broad-spectrum class of antibiotics that is more widely used in clinical practice. It can produce bactericidal effects by attacking the bacterial cell wall. Furthermore, Rokushima et al. suggested that cephaloridine might induce the expression of HSPA1B (44). Our study found that HSPA1B had a higher affinity for cephaloridine, which might laterally suggest that inflammatory factors play an important role in CAD. However, renal damage might result from high doses of cephaloridine (45). Cytochrome P450 bioactivation, mitochondrial dysfunction, lipid peroxidation, and other factors could be the causes.

Interleukin 17A (IL17A) is a proinflammatory cytokine secreted by activated T lymphocytes and belongs to the IL17 family (46). It is effective in inducing maturation of CD34+ hematopoietic precursors into neutrophils. Interleukin 17A receptor (IL17RA) binds to and functions with low affinity for IL17A. Through the secretion of IL17A, CD4+ T helper 17 (TH17) cells attract neutrophils and stimulate inflammatory responses (46). A positive correlation between IL17RA and neutrophils is exhibited in **Figure 6J**. **Figure 7B** also indicates that IL17RA strongly correlated with the chemokine family. IL17A mainly acts on non-immune cells, leading to generate chemokines such CXCL1, CXCL6, and CXCL8, which attracts neutrophils without directly acting on them. Receptors on neutrophils in vessels detect these chemokines, activating subsequent signals that facilitate neutrophil-directed movement (47). Furthermore, active neutrophils provide positive feedback on this procedure in order to improve recruitment. Neutrophil infiltration is inhibited, and chemokine expression is downregulated when IL17A expression is inhibited or when IL17A receptors (IL17RA) are damaged (48). There is a positive correlation among IL17RA, M0 macrophages, and CD 4+ T naive cells but negative with CD8+ T cells, as shown in **Figures 6K, L**. Polarizing effects of IL7RA on immune cells may have implications for CAD. Downregulating the IL7RA expression may prevent CAD progression by modulating the distribution of macrophages, neutrophils, and T-cell subtypes. Doxorubicin is a cytotoxic anthracycline broad-spectrum antibiotic commonly used as a tumor chemotherapeutic agent. Doxorubicin downregulates the basal phosphorylation of AMPK and downstream acetyl-CoA carboxylase and also induces apoptosis and autophagy (49). A study reported a high dose of doxorubicin used to create a myocardial injury model in wild-type (WT) and CRTH2 (a prostaglandin D2 receptor) receptor-deficient (CRTH2 KO) mice (50). The latter mice demonstrated a significant improvement in cardiac function and a decrease in mortality when compared with the former mice. Additional cardiac pathology sections of myocardial injured animals showed that CRTH2 KO mice had considerably lower cardiomyocyte apoptosis. These suggest that CRTH2 deficiency attenuates doxorubicin-induced myocardial injury in mice. Doxorubicin may induce an increased expression of IL17A protein, which can be strongly suppressed by thymoquinone (51). We found a total of four drugs through molecular docking. These four drugs are all commonly used in clinical practice, and by repurposing these, we may conduct several prospective studies to offer fresh approaches to the management of CAD. Despite the potential medications identified in our study, there are still unknowns waiting for exploration.

This work investigated and identified four diagnostic biomarkers, created a nomogram, explored the relationships with immune cells and immune families, and excavated the underlying drug therapies, all of which were helpful for further research on CAD. Nowadays, adjusting drug function has become a new strategy for disease treatment. With the deepening of disease mechanisms and the continuous improvement of therapeutic regimens, a variety of medications have been applied to the treatment of diseases. Based on this strategy, we can screen hub genes for targeted drugs with the aim of proposing a therapeutic approach to modulate poor prognosis.

Despite these meaningful findings, our study still has a few drawbacks. Firstly, we used only one database for bioinformatics excavating and found four diagnostic biomarkers. In order to be more relevant to clinical issues, we used the WGCNA package for initially searching potential markers. To avoid bias caused by samples’ selection, multiple datasets were utilized for validation. Secondly, although artificial intelligence is currently emerging and widely applied in both oncology and non-oncology fields, rational use of machine learning methods for screening of feature genes becomes particularly important. Here, we use both the RF and XGBoost methods to build the models. The RF runs quickly, is highly adaptive, and can produce more features. The XGBoost method is optimized with each screening round to lower the likelihood of overfitting, which somewhat offsets the drawbacks of the random forest algorithm. By complementing each other, hub genes were identified. Thirdly, information regarding the precise molecular pathways by which genes, miRNAs, and transcription factors affect these diseases is still lacking. Additional experimental and clinical research is required to confirm the function of the hub genes and the *in vivo* effectiveness of the potential therapeutic drugs that have been identified. The utilization of nomogram necessitates the collaboration and validation of more centers. Should the chance present itself, we will carry out multicenter prospective experiment in the future.

## 5 Conclusion

This investigation came to the conclusion that CSF3R, EED, HSPA1B, and IL17RA are the diagnostic biomarkers in CAD. The outcomes of this investigation also showed that immune reactions might be involved in the development and progression of CAD. Additionally, it was discovered that four hub genes had great relationships with a range of immune cell types. Immune-related molecules like those listed above are predicted to have a pivotal role in the emergence and progression of CAD. Additionally, it is probable that promising drugs will support the selection of immunotherapy targets and the improvement of immunomodulatory therapy for CAD patients.

## Data availability statement

Publicly available datasets were analyzed in this study. This data can be found here: https://www.ncbi.nlm.nih.gov/geo/query/acc.cgi?acc=GSE42148; https://www.ncbi.nlm.nih.gov/geo/query/acc.cgi?acc=GSE180081;.

## Ethics statement

Ethical approval was not required for the study involving humans in accordance with the local legislation and institutional requirements. Written informed consent to participate in this study was not required from the participants or the participants’ legal guardians/next of kin in accordance with the national legislation and the institutional requirements.

## Author contributions

XJ: Conceptualization, Data curation, Investigation, Methodology, Resources, Visualization, Writing – original draft, Formal Analysis, Validation. YL: Conceptualization, Formal Analysis, Methodology, Resources, Visualization, Writing – original draft, Data curation. ZL: Validation, Visualization, Writing – review & editing, Formal Analysis. HZ: Validation, Visualization, Writing – review & editing, Data curation. ZX: Project administration, Supervision, Writing – review & editing. DW: Supervision, Validation, Writing – review & editing, Project administration.
